# Land security and crop theft in rural Tanzania

**DOI:** 10.1371/journal.pone.0334273

**Published:** 2026-01-02

**Authors:** Nelson A. Ochieng, Ulrike Grote, Arnab K. Basu

**Affiliations:** 1 Institute for Environmental Economics and World Trade, Leibniz University Hannover, Hanover, Germany; 2 Department of Economics and Social Studies, Ardhi University, Dar es Salaam, Tanzania; 3 Charles H. Dyson School of Applied Economics and Management, Cornell University, Ithaca, New York, United States of America; ICAR-CCRI: ICAR Central Citrus Research Institute, INDIA

## Abstract

Is the risk of agricultural crime victimization linked to land titling and different land tenure modes? Drawing on a unique household survey from rural Tanzania, we find that farmers cultivating individually-owned plots are more likely to experience victimization, such as theft and vandalism of staple crops, compared to those operating on family-owned plots. Moreover, land titling has a negative effect on crime victimization. Our results remain robust under several empirical strategies, namely double lasso logistic regression and a linear probability model, as well as propensity score matching and an inverse probability-weighted regression. The findings offer policy relevant insights for enhancing land security and reducing agricultural crime in rural settings.

## Introduction

We draw attention to the relationships between land security and the incidence of agricultural crime victimization. Land security is proxied by tenure modes and land titling, while crime includes acts such as crop theft and vandalism in the fields. This nexus has received little attention, even though agricultural crime remains pervasive, with evidence suggesting a growing trend in developing countries [1–4]. It continues to cast a shadow over the discourse on land investment, agricultural productivity and food security. Existing studies document how agricultural crime victimization affects farmers and rural communities. Victims often experience reduced income due to stolen or damaged produce, lowered productivity from repeated offenses, and psychological consequences such as fear or stress [5–7]. These outcomes, in turn, undermine food security and discourage future investments in land or labour [3,8].

Previous studies have predominantly focused on the characteristics and incidence of rural crime victimization [9,10] and emphasize environmental and geographical factors, such as remoteness, sparse settlement, and low police presence, as key drivers of agricultural crime in the rural context [1]. Beyond these spatial dynamics, socio-economic stressors such as poverty, unemployment, and weather-related shocks further increase household vulnerability to crime by creating economic distress and strain within the community [2,8]. In such resource-scarce rural settings, this distress drives individuals to resort to opportunistic theft as a coping mechanism [7,11].

Moving past spatial and socio-economic factors, this paper pioneers an exploration of the relationships between agricultural crime victimization and land security within a rural setting. Far less attention has been given to institutional factors, particularly the role of land security in shaping exposure to agricultural crime. Land tenure arrangements and the presence or absence of formal land titles may influence both the attractiveness of farmland as a target and the owner’s ability to protect it. For instance, land without secure documentation may be more susceptible to unauthorized access or theft of standing crops, while titled land may empower owners to make protective investments or seek redress through formal institutions [12,13]. In addition to that, unclear land rights may lead to crop vandalism arising from land use conflicts between farmers and pastoralists competing for land and water resources [14,15]. Land policies that favour farmers, intensify these tensions. They prompt herders to seek grazing alternatives in farming areas and thereby increasing the likelihood of crop vandalism [16]. Despite this plausible link, the intersection between land security and agricultural crime victimization remains underexplored in the empirical literature.

Leveraging a unique dataset from rural Tanzania, we address two questions: (i) Is the risk of agricultural crime victimization likely to be higher under individual land ownership vis-à-vis family ownership, and (ii) does rural land titling deter the likelihood of being a victim of agricultural crime? We distinguish between individual landholding and family landholding. Individual landholding refers to land controlled by a single household, which has full discretion over its use, including the rights to sell it or use it as collateral. In contrast, family landholding denotes land collectively owned by related families. Within this arrangement, the land is typically subdivided into parcels allocated to different family heads based on mutual agreement or by the head of a clan. These parcels are used for activities such as non-permanent crop cultivation and grazing. However, decisions regarding alternative uses or sale usually require consultation and agreement among the families.

Tanzania offers a suitable case study on this issue as it has undergone several land tenure reforms, including a transition from a communal to an individualistic land ownership system and the introduction of land titling through the issuance of Certificates of Customary Rights (CCROs) by village councils [12]. At the same time, agricultural crime, which affected an estimated 29% of rural households in 2016 [9], has emerged as an increasingly widespread problem, ranking among the top concerns for the public [17].

In more detail, with this paper we contribute to two main strands of literature. First, we add to the small body of literature that explores the structural and institutional determinants of agricultural crime victimization. By examining how land tenure and titling relate to exposure to crop theft and vandalism, we expand current understanding of the conditions under which rural crime occurs. Second, we contribute to the extensive literature on land reforms [18–20] by offering new evidence on how different forms of landholding, individual versus family, and the issuance of land titles influence household vulnerability to crime.

Existing crime theories provide context for our geographic setting and empirical results. Criminal activity can be attributed to a combination of factors such as socio-economic conditions, geographical characteristics, cultural dynamics, and individual motivations. Three main theories are used to explain the prevalence of crime, particularly in rural areas: social disorganization theory, differential association theory, and routine activity theory. Social disorganization theory examines the influence of social disorganization on varying rates of criminal victimization and offending, focusing on community structure, social control and crime type [21,22]. In contrast, differential association theory emphasizes the role of socialization and peer interactions in shaping criminal behavior, particularly in tightly-knit rural areas [23,24]. Neither of these theories is applicable in our case as similar community structures and sociocultural norms exist in our survey areas of rural Tanzania. Instead, we draw on the routine activity theory which emphasizes the role of opportunity by highlighting the convergence of motivated offenders, suitable targets, and a lack of capable guardians in time and space [25,26]. In our study area, where limited police presence creates favourable conditions for criminal activity [9], we anticipate a higher rate affecting individual landowners who lack the collective oversight provided under a communal land tenancy arrangement [27].

While these criminological theories explain conditions that give rise to crime, we also draw on an economic perspective, the investment under uncertainty theory, to explain how individuals can respond to such crimes. This theory suggests that individuals are more likely to invest in protection or productivity-enhancing measures when property rights are secure and future risks are minimized [28]. In our context, such investments, enabled by land titling, can include fencing, boundary marking, or surveillance, which reduce the suitability of targets and enhance guardianship. In this way, the theory complements the routine activity theory by explaining the conditions under which individuals are willing to take steps that ultimately reduce criminal opportunities.

The remainder of the paper is organized as follows: the next section presents the data and methods used in the study. This is followed by the results, discussion, and finally conclusion.

## Data and methods

### Data

We used primary household data collected by Ardhi University, Dar es Salaam in collaboration with Leibniz University Hannover and the TUM Campus Straubing, from November 1 to December 15, 2018. The study received formal approval from the Research Ethics Committee of Ardhi University, Dar es Salaam, Tanzania. Household surveys were conducted in two regions in Tanzania: Dodoma and Morogoro, spanning the central and eastern parts of the country. Both regions are predominantly inhabited by small-scale farmers, but differ in terms of weather and socio-economic conditions. Dodoma is a semi-arid region where mainly millet, maize, groundnuts, sorghum, grapes, and sunflower are grown. In addition, livestock rearing plays an important role in the local economy. In contrast, Morogoro is a sub-humid region primarily known for the cultivation of maize, rice, vegetables, sesame, and legumes, with limited livestock farming. While both regions face persistent poverty, food shortages are more common in Dodoma, which also has a higher proportion of economically vulnerable households than Morogoro [29].

The districts of Chamwino in Dodoma and Kilosa in Morogoro were selected due to their agro-ecological conditions, which represent about 70–80 percent of the land-based farming systems in Tanzania [30]. Within each of these districts, three villages were selected: Ilolo, Ndebwe and Idifu in Chamwino, and Changarawe, Nyali and Ilakala in Kilosa. The villages were chosen based on similarity across various criteria such as market access, climatic conditions, dependence on rain-fed cropping systems, crop-livestock integration, and a relatively consistent village size of 800–1500 households. In each village, 128–146 households were randomly selected for interview, resulting in a total of 818 households for this study.

Prior to commencing data collection, verbal permission to conduct the household surveys was obtained from the Village Executive Officers in each study village and from the respective Ward Executive Officers. These officials are recognized within the Tanzanian local government structure as the legitimate representatives of the community. They were identified based on their administrative role and authority to grant research permissions at the village and ward level. Their verbal consent was obtained during introductory meetings, in which the study objectives and methods were explained. These leaders also facilitated household selection by helping to organize community members for participation. In addition, the informed consent was obtained verbally from participants, and the process was documented in accordance with the ethics committee’s requirement. Since no minors were directly interviewed, their information was gathered from their parents or guardians as necessary.

Table 1 below describes the variables used in our analysis. Crime victimization in the form of theft and vandalism of crops in the fields, is the dependent variable. Our main independent variables of interest are the land tenure mode and the possession of land title or certificate. For land tenure mode, we code land ownership into three distinct categories: family landholding, individual landholding, and rented-in land (by either a family or an individual). It is important to note that some households had multiple plots of land across the above three categories. In these cases, we consider the highest fraction of plots in a category to be the dominant form of tenure for the household.

**Table 1:** Description of the Variables.

Variable name	Description
Crime victimization	If household encountered agricultural crime victimization, including crop theft and vandalism in the fields, within the past 12 months.
Mode of tenure	Includes individual landholding, family landholding, and renting-in. With individual landholding, the owner has autonomous discretion over the use of land, including the ability to sell it or use it as collateral. In family landholding, the land is collectively owned by a group of related families with no autonomous discretion over the use or ownership transfer.
Land title	Possessing formal land ownership documents or certificates including title deeds, particularly for farmed plots.
Land conflict	Measures if there was any conflict related to the farm plot, including conflicts with neighbors, pastoralists, the government, private enterprises, or conflicts within the family.
Gender	Gender of household head.
Age	Age of household head.
Education (Years)	Number of years of education of household head.
Number of plots	Number of plots farmed by household head.
Time spent home by head (Months)	Number of months a household head stayed at his/her homestead within a year.
Average farm distance (minutes)	Average commuting time (in minutes) from homestead to the farm plot.
Household monthly income (US$ PPP 2010)	Monthly household income from various sources.
Membership	If a household is a member in any social or political groups in the village

### Methods

We treat our dependent variable (agricultural crime victimization) as a yes/no dummy and examine the association between land tenure mode, land titling and agricultural crime victimization via the double lasso logistic regression method. As a further robustness check, we use propensity score matching to assess the impact of tenure mode and titling on victimization. The stability and reliability of the treatment effect estimates from our propensity score matching model are then subjected to doubly robust estimation using the inverse probability weighted regression adjustment (IPWRA) method.

In addition to that, a minimal proportion of households were operating on rented-in farms (4%) in comparison to family and individual landowners. Consequently, we conduct tests to compare specific pairwise differences between the groups. The results indicate that there is no significant difference between individual landholding and rented-in landholding ($\chi^{\text{2}}$ = 0.002; p = 0.998). As a result, the two groups, individual landholders, and rented-in landholders, were merged during the empirical analyses and both treated as individual landholders.

#### Double lasso logistic estimation.

The double lasso analysis allows identification of the relevant covariates for the final estimation model and the set of confounding variables – land tenure mode (M) and possession of land title (T). It is now widely accepted that this two-stage analysis process, consisting of variable selection and coefficient estimation for the selected variables, helps mitigate bias and improve the accuracy of the model [31]. In the first stage, we estimate the confounding variables – land tenure mode (M) and possession of land title (T) – via the equations $M_{i}=\delta_{\text{0}}+\delta_{\text{1}}W_{i\text{1}}+\;\ldots +\delta_{k+\text{1}}W_{ik}+\varepsilon_{i}$ and $T_{i}=\delta_{\text{0}}+\delta_{\text{1}}W_{i\text{1}}+\;\ldots +\delta_{k+\text{1}}W_{ik}+\varepsilon_{i}$ respectively. In the second stage, we estimate $Y_{i}=\beta_{\text{0}}+\beta_{\text{1}}M+\beta_{\text{2}}T+\beta_{\text{3}}W_{i\text{1}}+\;\ldots +\beta_{k+\text{1}}W_{ik}+\varepsilon_{i}.$ The model aims to minimize the sum of error squares and introduces an additional penalty term $\lambda \sum_{k}\left|\beta_{k}\right|\;$in $Min[\sum {\left(Y_{i}-\beta_{\text{0}}+\beta_{\text{1}}M\;{+\;\beta }_{\text{2}}T\;+\beta_{\text{3}}W_{i\text{1}}+..+\beta_{k+\text{1}}W_{ik}\right)}^{\text{2}}+\lambda \sum_{k}\left|\beta_{k}|\right]$. The value of λ chosen shrinks the estimated regression coefficients towards zero.

We use a linear probability model (LPM) that incorporates the lasso-selected variables, confounders, and their interactions. The LPM model specification is written as


$$Y_{i}=\beta_{\text{0}}+\beta_{\text{1}}M+\beta_{\text{2}}T+\sum {\mathrm{\backslash nolimits}}_{k\in A}\beta_{k+\text{1}}W_{ik}\;+\varepsilon_{i}$$

Where: ${\text{Y}}_{\text{i}}$ is the dummy variable (crime victimization for household i), M and T represent mode of tenure and possession of land title, respectively, W_ik_ is the set of control variables including households’ socio-economic characteristics. The set A is the union of the variables estimated to have non-zero coefficients in the double lasso estimation.

To assess the robustness of the double-lasso findings, we also estimated ridge (L2) and elastic-net logistic regression models with tuning parameters selected via 10-fold cross-validation. Ridge regression applies L2 penalization, shrinking coefficients toward zero without performing variable selection, while elastic-net combines L1 (lasso) and L2 penalties to achieve both coefficient shrinkage and variable selection [32,33]. We compared the key predictors, coefficient magnitudes, and post-estimation linear probability results across these approaches to ensure that the substantive conclusions do not depend on any single regularization or variable-selection method.

#### Propensity score matching.

To check the validation of our results from the double lasso logistic regression and linear probability model, we use propensity score matching to assess the effect of land tenure mode and land titling on agricultural crime victimization. In this analysis, our treatment variables are land tenure mode and land title possession, while the outcome variable is victimization. The treatment group for tenure mode is family landholder, while the treatment group for land titling consists of households with land titles or certificates of customary rights (CCROs) for all their cultivated farm plots within the reference period.

We use two different matching algorithms: the nearest neighbour and the kernel density methods to pair treated and untreated observations for comparison. The former matches each treated individual with the nearest untreated individual based on the propensity score, while the latter uses kernel density estimation to estimate the propensity score distribution for both the treated and untreated observations and make the matches based on the overlap of the estimated distributions [34]. We thus calculate the treatment effect using $ATT=E\left(Y_{\text{1}i}|\tau_{i}=\text{1}\right)-E\left(Y_{\text{2}i}|\tau_{i}=\text{0}\right)$, where ATT is the average treatment effect on the treated. Here, E(Y_1i_|τ_i_ = 1) represents the expected outcome variable (Y) for individual i who received the treatment (τ_i _= 1), and E(Y_2i_|τ_i_ = 0) is the expected outcome variable (Y) for individual i who did not receive the treatment (τ_i_ = 0) but is similar to the treated group. E(Y_2i_|τ_i_ = 0) is thus the average outcome for the control group that matches the treated group based on the propensity scores. Details on the common support and balancing tests are presented in the Figs 1 and 2 in S1 File.

Since we lack a valid external instrument to address potential unobserved heterogeneity, we conduct a Rosenbaum bounds sensitivity analysis [35,36] to assess the extent to which hidden bias from unmeasured confounders would need to affect treatment assignment to overturn our conclusions. This allows us to evaluate the robustness of the estimated treatment effects beyond observed covariates.

#### Doubly robust estimation.

We employ doubly robust estimation using the inverse probability-weighted regression adjustment (IPWRA) method to assess the stability and reliability of the treatment effect estimates obtained from the propensity score matching analysis. By considering both matching and regression adjustment, we can better account for potential biases and potential confounders, resulting in more robust and reliable treatment effect estimates [37].

#### Model performance and diagnostics.

To assess model performance, we used different diagnostics tailored to our main models. For the linear probability model (LPM), predictive fit was evaluated using classification metrics, including accuracy, sensitivity, specificity, precision, and the Receiver Operating Characteristic (ROC) curve with its Area Under the Curve (AUC). For the propensity score matching (PSM) analyses, treated households were matched with untreated households based on predicted propensity scores from logit models. The validity of the matching process was assessed through examination of common support (overlap of propensity score distributions) and covariate balance between treated and control groups using standardized mean differences, median bias, and Pseudo-R squared, following Caliendo and Kopeinig [34]. These diagnostics ensure that the models provide a credible basis for estimating the relationships between land tenure, land titling, and agricultural crime victimization.

## Results

### Descriptive results

In Table 2, we observe that agricultural crimes, particularly crop theft and vandalism, are prevalent. In the past 12 months, 23% of households reported experiencing victimization on their farm plots at least once. On average, these households faced nearly three incidents (2.65), indicating a recurring pattern. Despite the frequency of these crimes, only 35% of victims adopted preventive measures, primarily in the form of guarding, neighbourhood watch, and installing locks. Notably, almost half (47%) of the victims did not report these crimes to the authorities.

**Table 2:** Comparison of agricultural crime types: theft and vandalism.

Variable name	Full sample (N = 188)		Theft (N = 69)		Vandalism (N = 119)		Mean difference (V-T)
	Mean	SD	Mean (T)	SD	Mean (V)	SD	
Frequency of occurrence	2.65	2.56	2.57	2.32	2.70	2.70	0.13
Case reported (yes = 1)	0.53	0.50	0.30	0.46	0.66	0.47	0.36***
Value lost (US$ PPP 2010))	84.9	130	42.1	65.0	109.7	151.0	67.5***
Measure taken (Yes = 1)	0.35	0.48	0.28	0.45	0.39	0.49	0.11
Crime re-occur (yes = 1)	0.07	0.26	0.06	0.24	0.08	0.28	0.02

SD is Standard Deviation; Significance level: 1% ***; 5%**, 10%*.

A clear discrepancy emerges when examining cases of victimization by agricultural crime, highlighting the disproportionate relationship between thefts and vandalism. As Table 2 shows, there were more victims of vandalism than of theft. Moreover, the average losses per victimization due to vandalism are significantly higher (USD 109.7), and they exceed those of theft (USD 42.1). This disparity is consistent with the reporting behaviour of farmers to local authorities, with more vandalism victims (66%) reported than theft (30%). Additionally, despite the implementation of various precautionary measures, crime incidents tend to persist after initial occurrences (7% recurrence), with no difference between theft and vandalism. The measures taken include guarding, which stood as the predominant preventive measure against theft and vandalism (65% of households that took precautions), followed closely by neighbourhood watch (25%). The installation of locks was relatively infrequent (10%), primarily used for farms integrated with homesteads and enclosed by thorny bushes or trees, featuring a lockable entrance gate.

Table 3 explains agricultural crime victimization in more detail based on various dimensions. For instance, more educated household heads are more likely to be victimized as compared to those with lower education levels. Note though that the average years of schooling is just over four years, meaning that many have not attended or completed primary education, which typically lasts seven years. Households on average indicate farming on two small plots with average size of two hectares.

**Table 3:** Summary statistics of agricultural crime victimization and land variables.

Variable name	Full sample (N = 818)		Victimized (N = 188)		Non-Victimized (N = 630)		Mean difference (B-A)
	Mean	SD	Mean (A)	SD	Mean (B)	SD	
Crime victimization (Yes = 1)	0.23	0.42					
Tenure modes							
Family owners	0.53	0.50	0.45	0.50	0.55	0.50	0.10**
Individual owners	0.44	0.50	0.51	0.50	0.42	0.49	−0.09**
Renters	0.04	0.19	0.04	0.20	0.03	0.18	−0.01
Land titling (yes = 1)	0.08	0.27	0.05	0.23	0.09	0.28	0.04*
Land Conflict (yes = 1)	0.09	0.29	0.17	0.38	0.07	0.25	−0.11***
Gender (male = 1)	0.74	0.44	0.74	0.44	0.74	0.44	−0.01
Age (years)	52.7	16.0	52.4	14.7	52.8	16.3	0.42
Education (years)	4.35	3.22	4.89	3.22	4.19	3.49	−0.70**
Number of farm plots	2.10	1.11	2.12	1.11	2.09	1.11	−0.04
Months spent at home by head	11.7	1.11	11.7	1.01	11.6	1.14	−0.10
Farm distance from home (minutes)	25.4	33.6	28.8	38.4	24.3	32.0	−4.44
Household monthly income (US$ PPP 2010))	181	246	159	182	187	262	27.4*
Membership	0.50	0.50	0.57	0.50	0.47	0.50	−0.9**

SD is the Standard Deviation; Significance level: 1% ***; 5%**, 10%*.

Shifting the focus to land-related variables, the majority (53%) of households carry out their agricultural activities on family-owned land, 44% on individually-owned land, and a small percentage (4%) on rented land (see Table 3). Additionally, when comparing victims and non-victims of crime, a lower proportion (45%) of family landowners were victims as compared to non-victims of crime (55%). Conversely, a larger proportion (51%) of individual landholders were victims compared with those who were not (42%). However, the proportion of victims was comparable among those who worked on rented land. Furthermore, the ownership rate of a land title is low (8% of the sample) with the proportion being lower among the crime victims than among non-victims. It is important to note that our survey did not capture victimization at the land parcel level. To establish a link between land (farm) titling and victimization at a household level, we define a household as “land-secure” if all of its farms have land certificates. This designation indicates that the household is not vulnerable to the risk of lacking a land certificate. Land conflicts also represent an important issue. A larger proportion (17%) of households who have experienced land conflicts are also victims of agricultural crimes.

Table 4 shows how different procedures of land acquisition relate to land titling across all reported land parcels, including farms and residential plots. In our study area, as in many other African countries, the most common method of acquiring land is through inheritance [38], accounting for 45.2% of all land parcels. This was followed by land acquisition through purchasing (21.4%), allocation by village authorities (11.4%), renting (7.8%), and by clearing forests or gift (14.2%). Notably, the likelihood that a land parcel has a formal title varies significantly by acquisition method. Among purchased parcels, 67.7% are titled compared to only 19.4% of inherited parcels. This reflects the more formalized procedures typically involved in land purchases, particularly when land is transferred to non-family members. In contrast, inherited parcels often remain undocumented due to customary practices and a lack of legal enforcement. This lack of documentation may expose landholders to a variety of land-related problems, including overlapping claims, boundary disputes, and land conflicts [13].

**Table 4:** Procedure of land acquisition and land titling.

Variable name	Full sample (N = 2,545)		With title (N = 325)		Without title (N = 2,220)		Mean difference (B-A)
	Mean	SD	Mean (A)	SD	Mean (B)	SD	
Inherited (yes = 1)	0.452	0.498	0.194	0.396	0.490	0.500	0.296***
Purchased (yes = 1)	0.214	0.410	0.677	0.468	0.146	0.353	−0.531***
Allocated by village (yes = 1)	0.114	0.318	0.040	0.196	0.125	0.331	0.085***
Cleared forest/gift (yes = 1)	0.141	0.349	0.061	0.241	0.153	0.360	0.092***

SD is the Standard Deviation; Significance level: 1% ***; 5%**, 10%*.

Regarding land use, crop cultivation remains an essential economic pillar in the region. Over time, there has been a significant transition in land use patterns, with many parcels originally classified as forest, abandoned cropland, or grassland being converted into actively used cropland and homesteads. This shift reflects both agricultural expansion and rural settlement growth. For detailed figures, see Table 11 in S1 Appendix in S1 File.

With regard to land conflicts, we find that the majority of cases involve pastoralists (77.2%), followed by neighbours (10.1%), and family members (6.0%), while the remaining 6.7% involve conflicts with the government, private enterprises and others. Since most villages do not have effective land use plans and in view of the changes observed, several conflicts have emerged between herders and farmers, particularly with the Maasai, most of whom are pastoralists from northern Tanzania [39,40]. Farmers’ experiences reported in Neubacher et al. [7] provide valuable context for understanding land-related victimization in rural Tanzania. In their focus group discussion, farmers emphasized that conflicts with the Maasai are the most common and often extend beyond simple grazing disputes and reflect deeper social and ethnic tensions within communities. These narratives illustrate that land conflicts are complex and multifaceted.

### Econometric results

We present results from the double lasso procedure, designed to estimate the effect of land tenure mode and land titling on household-level agricultural crime victimization. Table 5 reports the estimates from both a Linear Probability Model (LPM) and a Double Lasso (DL) logistic regression. These models incorporate covariates selected through cross-validation, ensuring robustness in variable selection and mitigating overfitting.

**Table 5:** Linear Probability Model and Double Lasso Logistic Regression of Tenure Mode and Land Titling on Crime Victimization.

Crime victimization (yes = 1)	Linear probability model using lasso selected covariates		Double lasso logistic regression model	
	Coefficients	Robust SE	Odds Ratio	Robust SE
Primary variables				
Tenure mode (*Family = 1)*	−0.057*	(0.029)	0.706**	(0.121)
Land titling (yes)	−0.163***	(0.052)	0.476**	(0.175)
Main effect covariates				
Age (years)	0.001	(0.001)		
Gender (male)	−0.002	(0.034)		
Land conflict (yes)	0.239***	(0.060)		
Number of farm plots	0.033	(0.021)		
Education (years)	0.025***	(0.009)		
Time spent home (months)	0.016	(0.013)		
Average farm distance (minutes)	0.001	(0.000)		
Household income	−0.000	(0.000)		
Membership (yes)	0.050*	(0.029)		
Interaction covariates				
Land titling X education	0.011	(0.013)		
Number of plots X education	−0.007**	(0.003)		
Constant	−0.094	(0.159)		
Number of observations	818		818	
Model fit and predictive performance				
R^2^	0.058			
F-test (p-value)	4.58 (0.000)			
Accuracy	0.593			
Sensitivity (Recall)	0.654			
Specificity	0.576			
Precision	0.315			
ROC/AUC	0.660	(0.022)		
Wald Chi^2^			9.49	
Prob>Chi^2^			0.0087	

Significance level: 1% ***; 5%**, 10%*; SE are Standard Errors.

The predictive fit of the LPM, summarized by accuracy, sensitivity, specificity, precision, and ROC/AUC, is reported at the bottom of Table 5. Using a cutoff equal to the sample prevalence of victimization (23%), the model correctly classifies 59% of households, with a sensitivity of 65% (correctly identifying victimized households) and a specificity of 58% (correctly identifying non-victims). Precision is modest at 31%, which is expected in contexts involving relatively rare events. The ROC analysis yields an AUC of 0.66 (95% CI: 0.62–0.70), indicating moderate discrimination ability and performance well above random classification. Overall, these diagnostics suggest that the model exhibits reasonable predictive capacity and provides an adequate basis for estimating the relationship between tenure security and crime victimization.

The results in Table 5 show consistent significance in both the LPM and DL estimation models, albeit with slight variations in the degree of significance for both tenure mode and land titling. This is primarily due to the additional reduction of the coefficient in the DL logistic model. The analysis reveals that individual landholders face a significantly higher risk (5.7% higher) of victimization compared to family landholders. Furthermore, households possessing land certificates are less likely to be a victim of crime (16.3% lower) than households without land certificates. In addition, variables such as land conflict, membership in social or political groups, and education level of the household head are positively associated with the likelihood of crime victimization.

Ridge and elastic-net logistic regression models, estimated with tuning parameters via 10-fold cross-validation, were used to assess the robustness of the double-lasso findings. As shown in Table 6, both approaches confirmed the main results obtained from the double-lasso procedure. The key predictors—tenure mode and land titling—remain statistically significant with coefficient directions consistent across methods. Post-estimation linear probability results indicate that family landholders have approximately a 5.6% lower probability of victimization, and households with land titles have an 11% lower probability of victimization. Although these effect sizes are slightly smaller than those estimated in the double-lasso model, the direction and statistical significance remain similar. Overall, the ridge and elastic-net specifications reinforce the robustness of the primary results, showing that the substantive conclusions do not depend on any single regularization or variable-selection method.

**Table 6:** Ridge and elastic-net penalized logistic regression models with post-estimation LPM effects.

Crime victimization (yes = 1)	Ridge	Post-estimation LPM	Robust SE	Elastic-net (alpha = 0.5)	Post-estimation LPM	Robust SE
Selected variables						
Tenure mode (*Family = 1)*	−0.053	−0.056*	(0.029)	−0.042	−0.056*	(0.029)
Land titling (yes)	−0.102	−0.111**	(0.047)	−0.073	−0.108**	(0.046)
Age (years)	0.000	0.001	(0.001)		–	–
Gender (male)	−0.000	−0.000	(0.034)		–	–
Land conflict (yes)	0.213	0.238***	(0.061)	0.195	0.225***	(0.061)
Number of farm plots	−0.001	−0.002	(0.015)	–	–	–
Education (years)	0.010	0.011**	(0.004)	0.008	0.011**	(0.004)
Time spent home (months)	0.014	0.015	(0.013)	0.008	0.016	(0.013)
Average farm distance (minutes)	0.000	0.001	(0.001)	0.000	0.001	(0.001)
Household income	−0.000	−0.000	(0.000)	−0.000	−0.000	(0.000)
Membership (yes)	0.045	0.047*	(0.030)	0.034	0.047	(0.029)
Constant	0.028	0.015	(0.166)	0.101	0.000	(0.151)
Number of observations	818	818			818	

Significance level: 1% ***; 5%**, 10%*; SE are Standard Errors.

To further validate these findings, Table 7 presents the average treatment effect on the treated (ATT) using propensity score matching. Both nearest neighbor and kernel matching methods yield consistent results. Model performance and matching validity are confirmed, with full diagnostics reported in Table 10 in S1 Appendix in S1 File. The findings indicate that households with individual landholding experience a 6% to 7% higher chances of being victimized compared to family landholders. However, land titling is associated with a significant decrease in household crime victimization, with a reduction ranging from 12% to 14%.

**Table 7:** Average Treatment effect on the Treated (ATT) for household crime victimization.

Variable	Nearest Neighbour Matching		Kernel Matching	
	ATT	SE	ATT	SE
Family landholding	−0.07**	0.03	−0.06**	0.03
Land titling	−0.14***	0.05	−0.12***	0.05

Significance level: 1% ***; 5%**, 10%* ATE is Average Treatment Effect, SE are Standard Errors.

Finally, Table 8 presents results from a Doubly Robust estimation using Inverse Probability Weighted Regression Adjustment (IPWRA). This approach also provides consistent evidence that individual landholders are 6% more likely to be victimized than family landholders. The situation regarding land titling mirrors that of the propensity score matching. The estimates align consistently, indicating a reduction in crime victimization associated with land titling of about 12%, which is statistically significant and comparable to the estimate obtained from the propensity score matching exercise.

**Table 8:** Average treatment effect for household crime victimization using IPWRA.

Variable	IPWRA	
	ATE	Robust SE
Family landholding	−0.06**	0.03
Land titling	−0.12**	0.05

Significance level: 1% ***; 5%**, 10%*. ATE is Average Treatment Effect, SE are Standard Errors.

To assess the sensitivity to unobserved confounding, we conducted a Rosenbaum bounds sensitivity analysis [35]. The analysis shows that the estimated effect of land titling and tenure modes on crime victimization are highly robust: an unobserved confounder would need to increase the odds of assignment to land titling or a specific tenure mode by a factor of at least 3 (Γ = 3) to nullify the treatment effect. The associated p-values (sig⁺ and sig⁻) remain at 0 across all values of Γ from 1 to 3, indicating strong robustness to moderate levels of unobserved bias. This suggests that the results are unlikely to be explained by moderate or even relatively strong hidden bias.

## Discussion

Our empirical results, based on both a double lasso logistic regression model and propensity score matching with inverse probability-weighted regression analysis, show that individual land ownership increases the probability of victimization by 7%. In contrast, official land titling tends to reduce the risk of victimization by nearly 14%. We also find that victimized individuals lose up to 11% of the value of their annual crop production. These findings point to the broader role of secure tenure systems and formal land administration frameworks in shaping rural safety.

Our finding that the likelihood of victimization is higher for an individual than for a family landholding, may reflect the erosion of informal protective mechanisms. Family landholding often involves shared responsibilities among kin, spatial proximity of plots, and stronger integration within local social networks [41]. These features reinforce mutual surveillance and collective land defence, serving as informal deterrents against agricultural crime. This observation is consistent with the literature highlighting unintended consequences of land reforms. Transition from a communal to individual landholding can lead to land fragmentation and the weakening of traditional norms such as labor sharing and joint safeguarding of agricultural resources [42]. In areas lacking effective policing and property rights enforcement, this creates conditions that may encourage criminal activity [26,27,43]. This finding aligns with the routine activity theory, which posits that crime occurs when a motivated offender encounters a suitable target without capable guardianship [25,26]. In this context, the shift from family to individual landholding may reduce informal surveillance, weakening ‘guardianship’ and thereby increasing vulnerability to crime.

Qualitative narratives from rural Tanzania vividly echo this erosion of communal protection. Participants in Neubacher et al. [7] described a pervasive sense of insecurity, expressing how a lifetime of vigilance leaves one unable to fully rest: “*That is, you sleep with your eyes open… you sleep for a few hours and then you wake up… and you thank God*.” Farmers recounted guarding their fields until dawn, underlining the tangible loss of informal guardianship with the breakdown of communal structures.

This underscores a fundamental tension in the transition from communal to individual landholding: while individual tenure may offer certain benefits, it can also weaken traditional communal protection systems that have long safeguarded rural landholders. Addressing these trade-offs is crucial for designing land policies that support both land security and community-based protection.

Our second finding, that land titling significantly reduces the risk of agricultural crime, suggests that it may serve as a partial remedy for the lack of effective policing during the transition from a communal to individual land tenure systems. Although direct links between land titling and reduced crime victimization are not widely studied, existing literature highlights the numerous benefits of land titling that help lower this risk. Land titling encourages long-term investments in security strategies [44–48] by providing greater security, clearer boundaries, legal recognition, and institutional visibility. These features reduce ownership ambiguity, improve landholders’ ability to assert control, and thus mitigate opportunistic crime. Without titles, farmers often hesitate to make substantial investments due to fear of potential losses from encroachment or redistribution of land by village chiefs or leaders [49].

In addition, land titling reduces the frequency of land conflicts [13,19] as well as the likelihood of crop vandalism [43,50]. It also promotes agricultural productivity through increased investment [46,51,52]. Recent studies further document these benefits in terms of tenure security, investment, productivity, and land conflict reduction [12,13,19]. Together, these effects reduce opportunities for agricultural crime by strengthening property rights and incentivizing investment. This finding also supports the routine activity theory, as land titling facilitates investments, such as fencing and boundary demarcation, that enhance guardianship and reduce target suitability [25,26]. It also aligns with Investment under Uncertainty Theory, which posits that secure property rights lower perceived risks and encourage protective investments [28].

## Conclusion

We have analysed the association between land security and agricultural crime victimization in rural Tanzania and find that the likelihood of victimization is significantly increased for individually vis-à-vis family-farmed plots. Possible mechanisms leading to this connection may include a lack of policing, conflict and a diminished role of labour sharing and community protection under the individual farming mode. Regardless of the exact mechanism, we show that land titling has a positive impact by reducing the risk of agricultural crimes by 12–14 percentage points.

Building on our results, the findings point to concrete policy implications. First, since individual landholders are more vulnerable to agricultural crime, and given the ongoing shift toward individualized farming, strengthening communal oversight is essential. This can be facilitated through existing cooperatives or informal farming groups that can help organize local protection and foster cooperation. In parallel, the significant crime-reducing effect of land titling highlights the need to expand access to affordable, transparent, and decentralized land titling services. Strengthening the legal recognition of land rights not only enhances tenure security but also encourages long-term investment in protective measures and sustainable land use.

Although not the focus of our analysis, several observations are worth noting that warrant further investigation. These include the lack of preventative measures (only 35% of households took preventive measures after victimization) and reluctance to report crimes (47% of households did not report the crimes) which are indicative of broader problems related to food security and enforcement of property rights.

Lastly, while we mitigate selection bias through the use of propensity score matching and IPWRA, the cross-sectional nature of our data limits our ability to draw strong causal inferences or observe changes over time. Future research using panel data and additional quasi-experimental strategies could further strengthen the identification of causal pathways between land security and agricultural crime victimization.

## Supporting information

S1 File Supplementary analyses and robustness checks. (PDF)

S2 File Inclusivity in global research questionnaire. (DOCX)
