# Supplementary material for: Land security and crop theft in rural Tanzania
Source: PLoS One. 2026 Jan 2;21(1):e0334273. doi: 10.1371/journal.pone.0334273 (PMC12758811; doi:10.1371/journal.pone.0334273)
Supplement: S1 File — (PDF) [file pone.0334273.s001.pdf]

# Appendix

**Table 9 Multicellularity test**

| Variable | VIF | 1/VIF |
|---|---|---|
| Tenure mode (*Family =1)* | 1.01 | 0.988115 |
| Land titling (yes) | 1.04 | 0.961026 |
| Age (years) | 1.20 | 0.832748 |
| Gender (male) | 1.13 | 0.888342 |
| Land conflict (yes) | 1.03 | 0.974682 |
| Number of farm plots | 1.11 | 0.901693 |
| Education (years) | 1.21 | 0.825187 |
| Time spent home (months) | 1.07 | 0.931703 |
| Average farm distance (minutes) | 1.03 | 0.967714 |
| Household income | 1.10 | 0.905515 |
| Membership (yes) | 1.05 | 0.949548 |
| **Man VIF** | **1.09** | |

# Propensity Score Matching

In conducting the estimation using propensity score matching, we matched the treated households with the untreated households based on the predicted propensity scores derived from logit models. It was essential to ensure a substantial overlap in the predicted propensity score distribution (common support) to guarantee the validity of the matching process (53). Fig 1 and 2 presents the propensity score distribution for both the treated and untreated groups, specifically focusing on our treatment variables of land titling and land tenure mode, respectively.

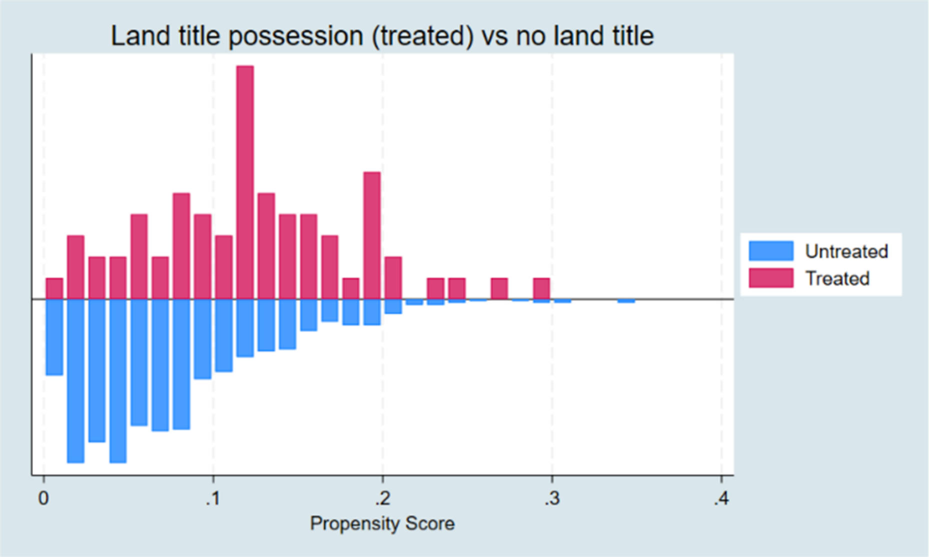


**Fig 1.** Propensity score distributions for land title possession (treated) versus no land title. The distributions are shown for two groups: untreated (blue) and treated (red).

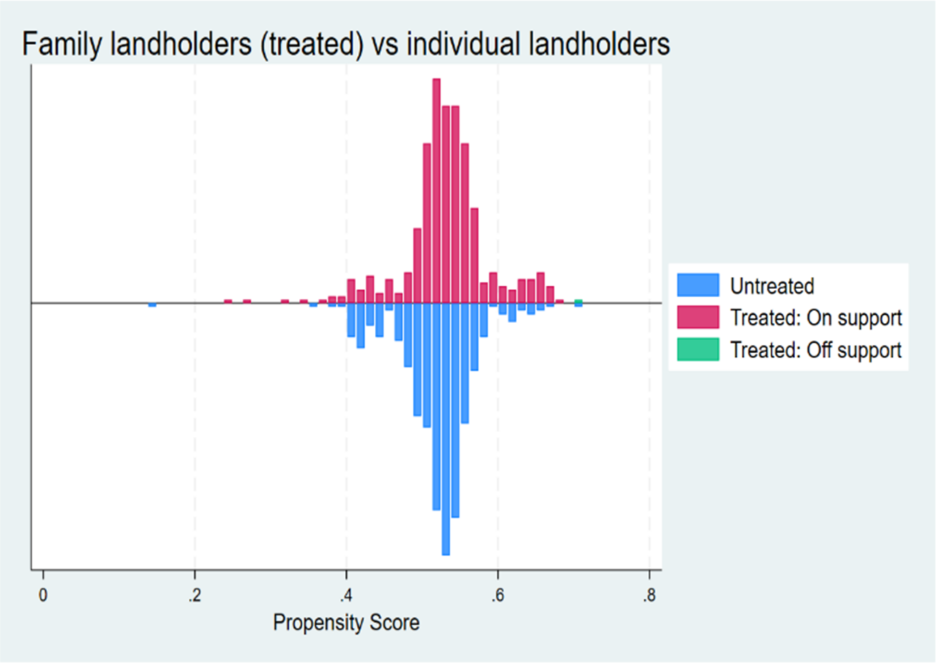


**Fig 2.** Propensity score distributions for family landholders (treated) versus individual landholders. The distributions are shown for three groups: untreated (blue), treated (on support, red), and treated (off support, green)

Furthermore, we assessed the efficiency of the matching procedure by performing the balancing tests as outlined by Caliendo and Kopeinig (34). The results

of these tests are presented in Table 10, revealing that, following the matching process, the average bias for land tenure mode decreased to 3.1% and for land titling, it decreased to 2%. Furthermore, the median biases for both variables also showed a significant reduction. Moreover, the matching process led to a significant decrease in the pseudo $R^2$, indicating an improved fit of the model. These findings suggest that the matching process successfully equalized the distributions between the treated and untreated groups (53).

**Table 10 Balancing tests before and after matching**

| Variable | Nearest Neighbour Matching | | Kernel Matching | |
|---|---|---|---|---|
| | Unmatched | Matched | Unmatched | Matched |
| Land tenure mode | | | | |
| Mean bias | 5.5 | 3.1 | 5.5 | 2.0 |
| Median bias | 4.2 | 2.7 | 4.2 | 1.5 |
| Pseudo $\boldsymbol{R}^2$ | 0.009 | 0.003 | 0.009 | 0.002 |
| Land titling | | | | |
| Mean bias | 17.7 | 4.1 | 17.7 | 15.3 |
| Median bias | 15.3 | 4.1 | 2.3 | 1.9 |
| Pseudo $\boldsymbol{R}^2$ | 0.077 | 0.005 | 0.077 | 0.002 |

# Transition in land use patterns

**Table 11 Land use during acquisition and the current use**

| Main land use | During acquisition (N=2,536) % | Current use (N=2,536) % |
|---|---|---|
| Cropland in use | 53.4 | 70.7 |
| Abandoned cropland | 7.73 | 0.24 |
| Forest | 20.4 | 0.08 |
| Grassland | 3.31 | 0.12 |
| Homestead | 14.3 | 28.0 |
| Other use | 0.91 | 0.91 |
